# Which Should Be Used First for ALK-Positive Non-Small-Cell Lung Cancer: Chemotherapy or Targeted Therapy? A Meta-Analysis of Five Randomized Trials

**DOI:** 10.3390/medicina55020029

**Published:** 2019-01-29

**Authors:** Yen-Chien Lee, Chung-Cheng Hsieh, Yen-Ling Lee, Chung-Yi Li

**Affiliations:** 1 Department of Oncology, Tainan Hospital, Ministry of Health and Welfare, Tainan 70043, Taiwan; yc_lee@post.harvard.edu (Y.-C.L.); yenpig8291@gmail.com (Y.-L.L.); 2 Department of Internal Medicine, National Cheng Kung University, Tainan 704, Taiwan; 3 Department of Molecular, Cell and Cancer Biology, University of Massachusetts Medical School, LRB#427, 364 Plantation Street, Worcester, MA 01605, USA; chung-cheng.hsieh@umassmed.edu; 4 Department of Public Health, College of Medicine, National Cheng Kung University, Tainan 704, Taiwan; 5 Department of Public Health, College of Public Health, China Medical University, Taichung 40402, Taiwan

**Keywords:** ALK inhibitor, chemotherapy, non-small-cell lung cancer, progression-free survival, overall survival

## Abstract

*Background and objectives:* Targeted therapy is widely used in the era of precision medicine. Whether the sequence in which targeted therapy and chemotherapy are performed matters, is however not known. We examined the impact of the sequential treatment of targeted therapy and chemotherapy among advanced anaplastic lymphoma kinase (ALK), non-small cell lung cancer (NSCLC) patients. *Materials and Methods:* Randomized controlled trials comparing the use of ALK inhibitors with chemotherapy were included in this meta-analysis. We estimated the hazard ratios (HRs) and 95% confidence intervals (CI), for progression-free survival (PFS) and overall survival (OS) from a random effects model. Two-sided statistical tests were used to determine the significance of these estimates. *Results:* In five eligible studies (1404 patients), ALK targeted therapy, in comparison with chemotherapy, had a significantly higher PFS (HR = 0.48; 95% CI, 0.42–0.55), but not significantly higher OS (HR = 0.88; 95% CI, 0.72–1.07). Crossover from chemotherapy to ALK inhibitors was allowed after progression in all trials. The sensitivity analysis of the use of ALK inhibitors as either the first- or second-line treatment, showed improvements in PFS but not in OS. *Conclusions:* Our results indicate that using targeted therapy first improved PFS, but that the sequence in which the treatments were performed did not cause a significant difference in overall survival.

## 1. Introduction

Lung cancer continues to be a leading cause of cancer-related deaths globally. Over the last decade, advances in precision medicine for non-small-cell lung cancer (NSCLC) have spurred the development of successful targeted therapies and have changed treatment guidelines. Anaplastic lymphoma kinase (ALK)-positive (ALK+) NSCLC represents 3–5% of all NSCLC patients [1]. Traditionally, chemotherapy has been the standard treatment before targeted therapies. It also might represent the last resource after the failure of targeted therapy treatment. In a retrospective analysis of patients with advanced ALK-positive NSCLC, crizotinib therapy is associated with an improved survival rate in comparison with crizotinib-naïve controls [2]. In current studies, crossover is usually allowed for ethical considerations. Whether the delayed use of targeted therapy with ALK inhibitors causes harm remains to be explored. In areas where ALK inhibitors needs to be self-paid, delayed targeted therapy with ALK inhibitors might reduce the economic burden on patients and might not affect the overall survival, if the sequence in which the therapies are performed is proven to make no difference.

This meta-analysis examines whether having targeted therapy as the first- or second-line of therapy affects either progression-free survival (PFS) or overall survival (OS), by pooling evidence from the currently available randomized controlled trials.

## 2. Methods

### 2.1. Study Selection

We conducted a review of MEDLINE (EBSCOhost) and PubMed up to 7 May 2018, and searched the databases using the keywords “ALK” and “lung cancer”, without limitation. We also hand-searched the relevant references. The potentially relevant studies were retrieved. For eligible studies that had multiple reports, only the most recent or complete publication was included.

The goal of the analysis was to examine the effects of exposure to chemotherapy or ALK inhibitors on progression-free survival and overall survival. Our analysis included only studies that fulfilled these criteria: (1) randomized phase III prospective trials in lung cancer patients, and (2) randomization in assigning patients to chemotherapy or ALK. Two reviewers (YCL and YLL) independently examined the title and the abstract of the publications identified by the search strategy. The full texts of all the potentially relevant publications were retrieved. A five-point Jadad ranking system [3] on randomization, double-blinding, and withdrawals was used to assess the quality of the study.

### 2.2. Data Extraction

We followed the Preferred Reporting Items for Systematic Reviews and Meta-Analyses (PRISMA, http://www.prisma-statement.org) statement for data extraction. The information that was extracted from each study included the publication year, the name of the first author, the trial type, the patient number, the median PFS, and the median OS.

### 2.3. Statistical Analysis

The heterogeneity among the trials was assessed using Cochrane’s Q statistic, and the degree of heterogeneity was quantified by the I^2^ statistic, which estimated the total percentage of variation across studies that was due to heterogeneity rather than to chance. A random-effects model was used to obtain a summary of the hazard ratios (HRs) and 95% confidence intervals (CI). The statistical significance was set at the two-sided *p*-value of <0.05. We used the Stata software (version 12.0, College Station, TX, USA) to perform all of the statistical analyses.

## 3. Results

### Search Results

**Figure 1:**
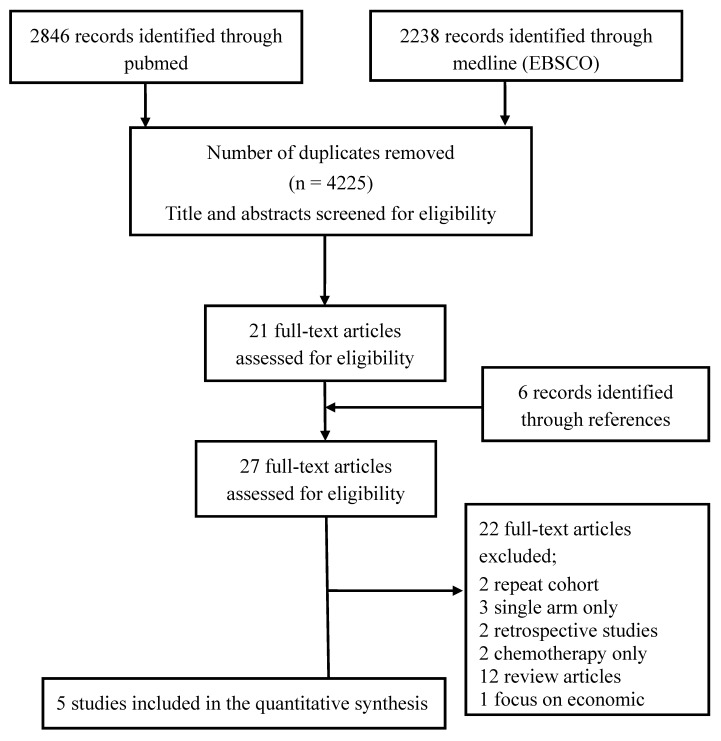
Selection of studies in the meta-analysis.

From a review of 4225 total titles or abstracts, 27 full articles were retrieved. Of these, five articles satisfied the inclusion criteria [1,4,5,6,7] and were therefore included in the analysis, see Figure 1. The reasons for excluding the other 21 articles were that they had a nonrandomized design (two articles), that they were a secondary publication (two articles), that the study was single-arm only (three articles), that their analysis was retrospective (two articles), that economics was the main focus of the article (one article), and that they were review articles (12 articles), see Figure 1. Table 1 displays the characteristics of the five studies.

In total, the five studies included 1404 patients, 721 of which were assigned to ALK inhibitors, while 683 were assigned to control arms, at random. The significance of the imbalance between the two groups was due to the unbalanced design, introduced by Novello et al. [4], where patients were randomized at a ratio of 2:1 to receive alectinib or chemotherapy. Cross-over after chemotherapy failure was allowed in all studies, but the inverse was not mentioned. The median age of the patients was 55. The brain metastasis status was balanced among all the studies. The other clinical characteristics of the patients are summarized in Table 1. All of the studies were open-label, phase 3 trials, so two points were deducted for not being double-blinded on the five-point Jadad scale, which was used to assess the quality of the studies, as seen in Table 2. Only two of the studies scored 3, two studies scored 2, and one study scored 1.

**Figure 2:**
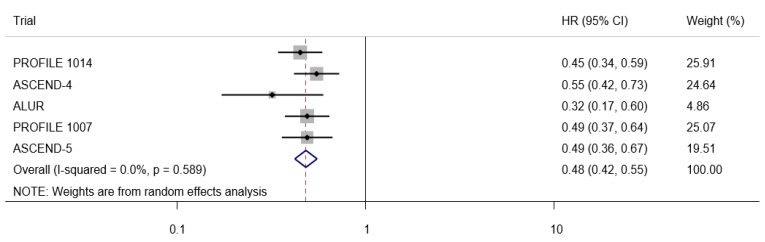
Forest plot of anaplastic lymphoma kinase (ALK) inhibitor versus chemotherapy on progression-free survival (PFS).

**Figure 3:**
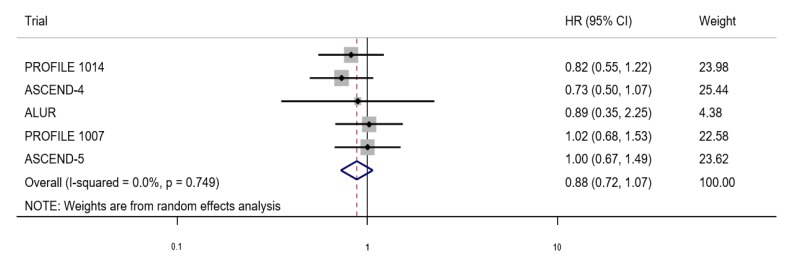
Forest plot of ALK inhibitor versus chemotherapy on overall survival (OS).

In comparison with chemotherapy, treatment with ALK inhibitors was associated with a significant reduction in PFS (HR = 0.48, 95% CI: 0.42–0.55), as seen in Figure 2, but not in OS (HR = 0.88, 95% CI: 0.72–1.07), as seen in Figure 3. No significant heterogeneity was found. In a sensitivity analysis, using the data for first-line ALK targeted therapy from two trials [1,5], the pooled HR for PFS was 0.50 (95% CI, 0.41–0.60) and 0.77 (95% CI, 0.59–1.02) for OS. In the three trials that had data on ALK targeted therapy as the second-line treatment [4,6,7], the pooled HR was 0.47 (95% CI, 0.39–0.57) for PFS and 1.00 (95% CI, 0.76–1.31) for OS. Again, no significant heterogeneity was observed.

## 4. Discussion

While chemotherapy has been shown to be effective in ALK-positive lung cancer patients, [8] and remains a viable option in patients with ALK translocations, it has been reported that chemotherapy-only treatment was inferior to all ALK inhibitor subgroups [9]. In this meta-analysis, we sought to learn whether delaying the use of ALK inhibitors in a combined chemotherapy–ALK inhibitor treatment, affects the overall survival in ALK-positive patients. Our analysis showed that whether chemotherapy was the first-line or targeted treatment with ALK inhibitors was the first-line therapy did not affect the overall survival of ALK-positive NSCLC patients. Cross-over from chemotherapy to the use of ALK inhibitors was allowed, following progression in all trials. However, none of the trials mentioned cross-over from ALK inhibitors to chemotherapy. Targeted therapy was effective in prolonging PFS, either as a first- or a second-line treatment. Because of the nature of the treatment involved and the treatment assignment (i.e., investigator preferences), effective blinding would have been difficult, therefore all of these studies were open label. Due to this limitation, we pooled all of the first-, second-, and third-line randomized studies together. The sensitivity analysis of first-line only or second-line only studies, still showed that targeted therapy first improved PFS but not OS. Therefore, there was no difference in overall survival between the use of targeted therapy or chemotherapy as the first choice. However, there are now at least three generations of ALK target inhibitors and the best sequence among these inhibitors remains unknown.

The most frequent site of relapse in patients receiving crizotinib is the central nervous system (CNS). In the ALEX trial [10], 12% of patients in the alectinib group had a CNS progression event, as compared with 45% in the crizotinib group (cause-specific HR 0.16; 95% CI, 0.10–0.28; *p* <0.001). Whether decreased brain metastasis translates into prolonged overall survival remains to be explored. Also, secondary ALK mutations are more common after treatment with second-generation ALK inhibitors [11]. The proportion of new brain metastasis under chemotherapy treatment was close to that of ceritinib, a second-generation inhibitor. In the ASCEND-5 trial [7], 62% of patients in the ceritinib group who had no brain metastases at baseline progressed, with most of the progressions outside of the brain (85%). In the chemotherapy group, among those without brain metastasis at baseline, 68% progressed. Among the patients with progression, 10% had intracranial progression only, 82% had extracranial progression only, and 8% had both. Determining whether the patients with brain metastasis survived less or had a poorer quality of life needs further investigation.

Multiple variants of the echinoderm microtubule-associated protein-like 4 (EML4)-ALK have been reported on, with V1, V2 and V3a/b as the most common. The mutations result in the constitutive ligand-independent activation of the downstream Ras/mitogen-activated protein kinase, the (MAPK)/extracellular signal regulated kinase (ERK), the PI3K/AKT and Janus kinase 3 (JAK3)/signal transducer and activator of transcription 3 (STAT3) [12]. The mechanisms of resistance to crizotinib include the ALK-independent mechanism (50%), ALK mutation (31%), ALK amplification (13%), or both ALK mutation and amplification (6%) [13]. Second-generation inhibitors (e.g., ceritinib, alecitinb and brigatinib) are generally effective, whether crizotinib-resistant or not, but cause a higher frequency of one mutation, ALK^G1202R^ [11]. We do not know if there is a tradeoff between using second-generation inhibitors, causing more ALK^G1202R^ mutations, and decreasing the use crizotinib, which might affect the overall survival. Third-generation lorlatinib is active against the ALK resistance mutations that developed against second-generation ALK inhibitors [11]. However, cell lines without ALK resistance mutations are resistant to lorlatinib [11]. Should we select targeted therapy, using the same rule as antibiotic treatment, by treating resistant bacteria at very low antibiotic concentrations with lower potency, and save the more potent inhibitors as the last resort? If this is true, we should use first-generation ALK inhibitors first. Or, alternatively, should the more potent drugs be used in order to prolong overall survival?

This study has some limitations. Among the five studies, three have not yet reported more mature data on OS. Also, we grouped the first-, second- and third-line studies together. Our results have shown that at least first-line treatment of ALK-positive NSCLC with chemotherapy, as the Taiwan national health insurance policy dictates, would not decrease OS as long as targeted therapy with ALK inhibitors is available as the second-line treatment.

## 5. Conclusions

The choice of the first-line treatment for ALK-positive, non-small cell lung cancer needs to take into account cost–benefit considerations and the patient-reported quality of life, as the treatment sequence did not cause a significant difference in overall survival.

## Figures and Tables

**Table 1:** Characteristics of the clinical trials.

Trials, Year	Setting	Regiment	Patient No.	Age (Median)	Cross-Over (%)	Initial Brain Meta (%)	Follow-Up Median Time (m)	PFS (m)	OS (m)
PROFILE 1014, 2014 [1]	First-line	Crizotinib vs. PEM + cisplatin	172 171	52 54	Yes (70%)	26 27	17.4 16.7	10.9 7	17.4 16.7
ASCEND-4, 2017 [6]	First-line	Ceritinib vs. PEM + platinum	189 187	55 54	Yes ((105/187)56%)	31 33	NA NA	16.6 8.1	NR 26.2
PROFILE 1007, 2013 [5]	Second-line	Crizotinib vs. PEM or TXT	173 174	51 49	Yes ((112/174)64%)	35 34	12.2 12.1	7.7 3.0	NR NR
ALUR, 2018 [4]	Two prior lines, crizotinib, platinum-based doublet	Alectinib vs. PEM or TXT	72 35 (2:1 block)	55.5 59	Yes (70.6%)	65.3 74.3	6.5 5.8	7.1 1.6	12.6 NR
ASCEND-5, 2017 [7]	1 or 2 chemotherapy, and crizotinib resistance	Ceritinib vs. PEM or TXT	115 116	54 54	Yes (64.7%)	57 59	19.7	5.4 1.6	18.1 20.1

In this table, NR indicates not reached; PEM indicates pemetred; and TXT indicates docetaxel. C/T indicates chemotherapy.

**Table 2:** Jadad scale analysis of controlled trials.

Trials, Year	Was the Study Described as Randomized?	Method to Generate the Sequence of Randomization was Described and Appropriate	Was the Study Described as a Double Blind?	Method of Double Blinding was Described and Appropriate	Was There a Description of Withdrawal and Dropouts?	Score
PROFILE 1014, 2014 [1]	*	-	-	-	*	2
ASCEND-4, 2017 [6]	*	*	-	-	*	3
PROFILE 1007, 2013 [5]	*	-	-	-	*	2
ALUR, 2018 [4]	*	-	-	- (note)	-	1
ASCEND-5, 2017 [7]	*	*	-	- (note)	*	3

Note: Open-label, blinded independent review committee.
